# Scleredema associated with immunoglobulin A-κ smoldering myeloma: a case report and review of the literature

**DOI:** 10.1186/s13256-019-2072-1

**Published:** 2019-05-14

**Authors:** B. S. D. P. Keragala, H. M. M. T. B. Herath, G. H. D. C. Janappriya, B. S. Dissanayaka, S. C. Shyamini, D. P. Liyanagama, Thanushah Balendran, S. R. Constantine, C. N. Gunasekera

**Affiliations:** National Hospital of Sri Lanka, Colombo, Sri Lanka

**Keywords:** Scleredema, Smoldering myeloma, Monoclonal gammopathy

## Abstract

**Background:**

Scleredema is a rare sclerodermoid skin condition characterized by diffuse symmetrical thickening of the upper part of the body. Its association with monoclonal gammopathy and myeloma was recently described; very few cases have been reported to date.

**Case presentation:**

A 66-year-old Sri Lankan woman who had been followed in a dermatology unit for 34 years with diffuse systemic sclerosis presented with an acute exacerbation of the skin disease. Absence of Raynaud’s phenomenon; sclerodactyly; characteristic lung, gastrointestinal, and cardiac involvement of systemic sclerosis; and repeatedly negative antinuclear antibodies test results led to reevaluation for the possibility of scleredema. Skin biopsies from four body sites showed normal epidermis and thickened reticular dermis with swollen collagen bundles separated from one another by clear spaces, resulting in fenestration. The skin appendages were not atrophied or bound down. Alcian blue staining showed interstitial mucin deposition. Serum protein electrophoresis demonstrated an abnormal monoclonal band in the β-region with a paraprotein level of 8.9 g/dl. Immunofixation showed an abnormal band in the γ-region consisting of immunoglobulin A and κ. Bone marrow biopsy revealed abnormal monoclonal plasma cells (15%) with multinuclearity. There was no evidence of end organ damage, and whole-body magnetic resonance imaging did not reveal any evidence of bone involvement. The patient’s diagnosis was revised as scleredema type 2 associated with IgA-κ, and she was referred to a hemato-oncologist for chemotherapy, which led to significant improvement in the skin condition.

**Conclusions:**

Scleredema is a rare disorder that has an enigmatic, rare association with monoclonal gammopathy. Dermatologists should be aware of this rare but important association.

## Introduction

Scleredema is rare sclerodermoid skin disease characterized by symmetrical diffuse woody induration of the upper part of the body owing to thickened dermis and excessive dermal mucin deposition. Though the commonest association of it is diabetes mellitus (type 3) [1], scleredema has been reported to occur with a history of an antecedent infection (type 1) and many other systemic diseases. Of them, monoclonal gammopathy was a recently described unusual association (type 2) with unknown significance. A high degree of suspicion is needed to differentiate scleredema from scleroderma when scleredema has a chronic course with generalized involvement. Owing to the rarity of the disease and subtle differences in the skin manifestation, histopathological assessment with mucin staining is invaluable in doubtful instances.

We report a case of a patient with long-standing widespread scleredema associated with immunoglobulin A-κ smoldering myeloma, which was misdiagnosed as scleroderma for many years. Only a few case reports are available in the literature on scleredema associated with myeloma; to the best of our knowledge, this is the first patient to be reported with scleredema who was diagnosed with smoldering myeloma of IgA-κ. This case report highlights the importance of awareness of scleredema because it is rare and can be misdiagnosed and, if diagnosed, it can be treated. We also include a detailed literature review.

## Case presentation

A 66-year-old Sri Lankan woman who had been followed in a dermatology unit for 34 years for diffuse systemic sclerosis presented to our institution with an acute exacerbation of the skin disease. She was treated with corticosteroids and cyclophosphamide pulses and subsequently with mycophenolate mofetil for the skin condition. She did not have any other past medical or family history of systemic diseases, chronic infections, malignancies, or genetic diseases. She was a housewife, was unemployed, and was not exposed to any indoor or outdoor toxins, chemicals, or radiation. She was a nonsmoker and nonalcoholic.

**Fig. 1:**
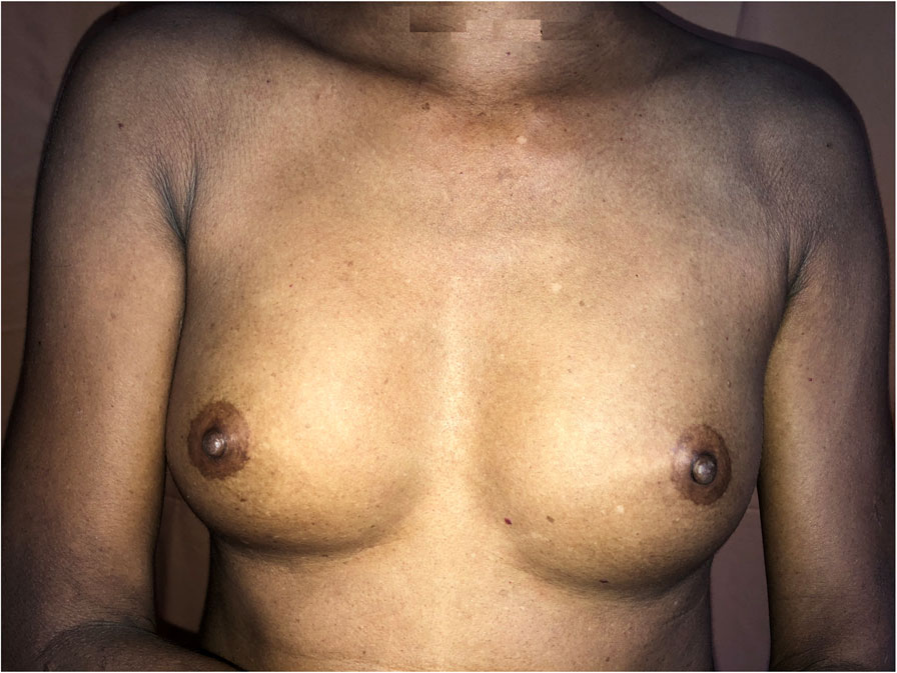
Widespread thickening of the skin predominantly involving the trunk and proximal extremities

**Fig. 2:**
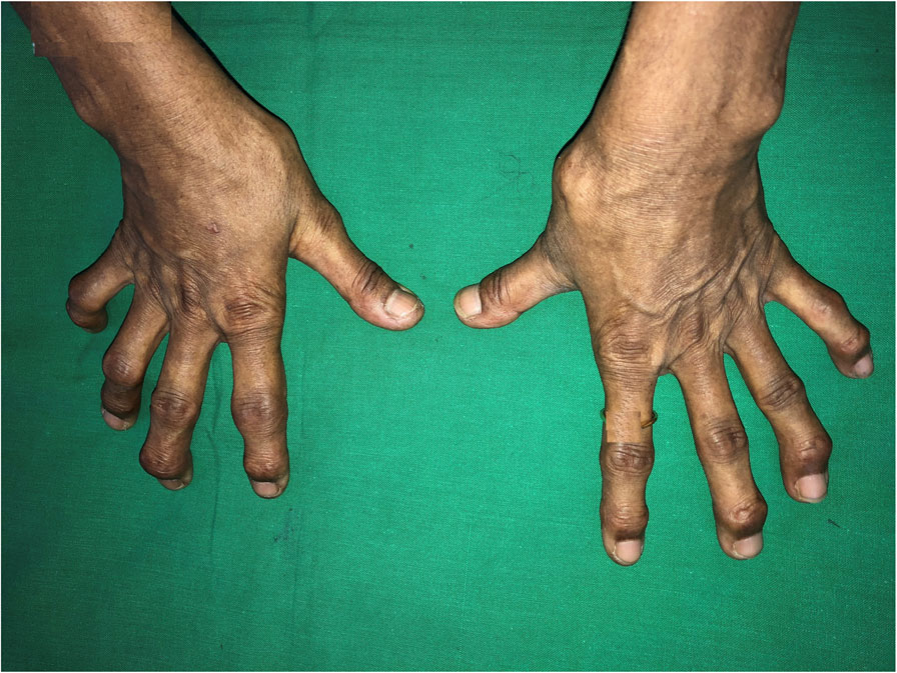
The patient did not have sclerodactyly but had deformities in keeping with osteoarthritis

On examination, she had widespread thickening of the skin predominantly involving the trunk and proximal extremities (Fig. 1). She did not have sclerodactyly, but she had deformities in keeping with osteoarthritis (Fig. 2). She denied cold-induced episodic acral bluish discoloration suggestive of Raynaud’s phenomenon. She was not febrile, and the result of her general examination was normal without pallor, cyanosis, clubbing, lymphadenopathy, or bilateral ankle edema. Her respiratory and cardiovascular examination results were normal with a heart rate of 82 beats per minute and a blood pressure of 130/80 mmHg. The result of her neurological examination was normal with normal funduscopy without any cranial neuropathy or peripheral neuropathy. Repeated echocardiography did not reveal any evidence of pulmonary hypertension. Upper gastroduodenoscopy did not show reflux disease. Radiographically, there was no evidence of interstitial lung disease. The patient’s autoimmune antibody profile (antinuclear antibody, anti-double-stranded DNA, perinuclear antineutrophil cytoplasmic antibodies, cytoplasmic antineutrophil cytoplasmic antibodies, anti-Smith antibody, anti RO and anti-LA, antitopoisomerase antibody, anticentromere antibody, and complements) was persistently negative, and her full blood count, urine full report, and renal and liver function were normal (Table 1). Absence of Raynaud’s phenomenon; sclerodactyly; characteristic lung, gastrointestinal, and cardiac involvement of systemic sclerosis; and repeatedly negative antinuclear antibody test results lead us to reevaluate the patient for the possibility of scleredema.

**Table 1:** Full blood count, liver function test, and serum electrolyte results

Investigation	Value	Normal range	Comment
WBC	9.32 × 10^3^/μl	4–10	Normal
Lymphocytes	2.17 × 10^3^/μl	0.8–4	Normal
Serum creatinine	0.9 mg/dl	60–120	Normal
Serum potassium	3.4 mmol/L	3.5–5.1	Normal
AST	27 U/L	10–35	Normal
Albumin	38 g/L	35–45	
Alkaline phosphatase	104 U/L	100–360	Normal
Ionized calcium	1.21 mmol/ L	1.0–1.3	Normal
Amylase	68 U/ L	22–80	Normal
Neutrophils	6.09 × 10^3^/μl	2–7	Normal
Platelets	277 × 10^3^/μl	150–450	Normal
Serum sodium	138 mmol/L	135–148	Normal
			Normal
ALT	20 U/L	10–40	Normal
INR	1.26		Normal
Serum magnesium	1.7 mg/dl	1.7–2.7	Normal
Troponin I	< 0.1 ng/ml	< 0.5	Normal

*Abbreviations: ALT* Alanine aminotransferase, *AST* Aspartate aminotransferase, *INR* International normalized ratio, *WBC* White blood cells

**Fig. 3:**
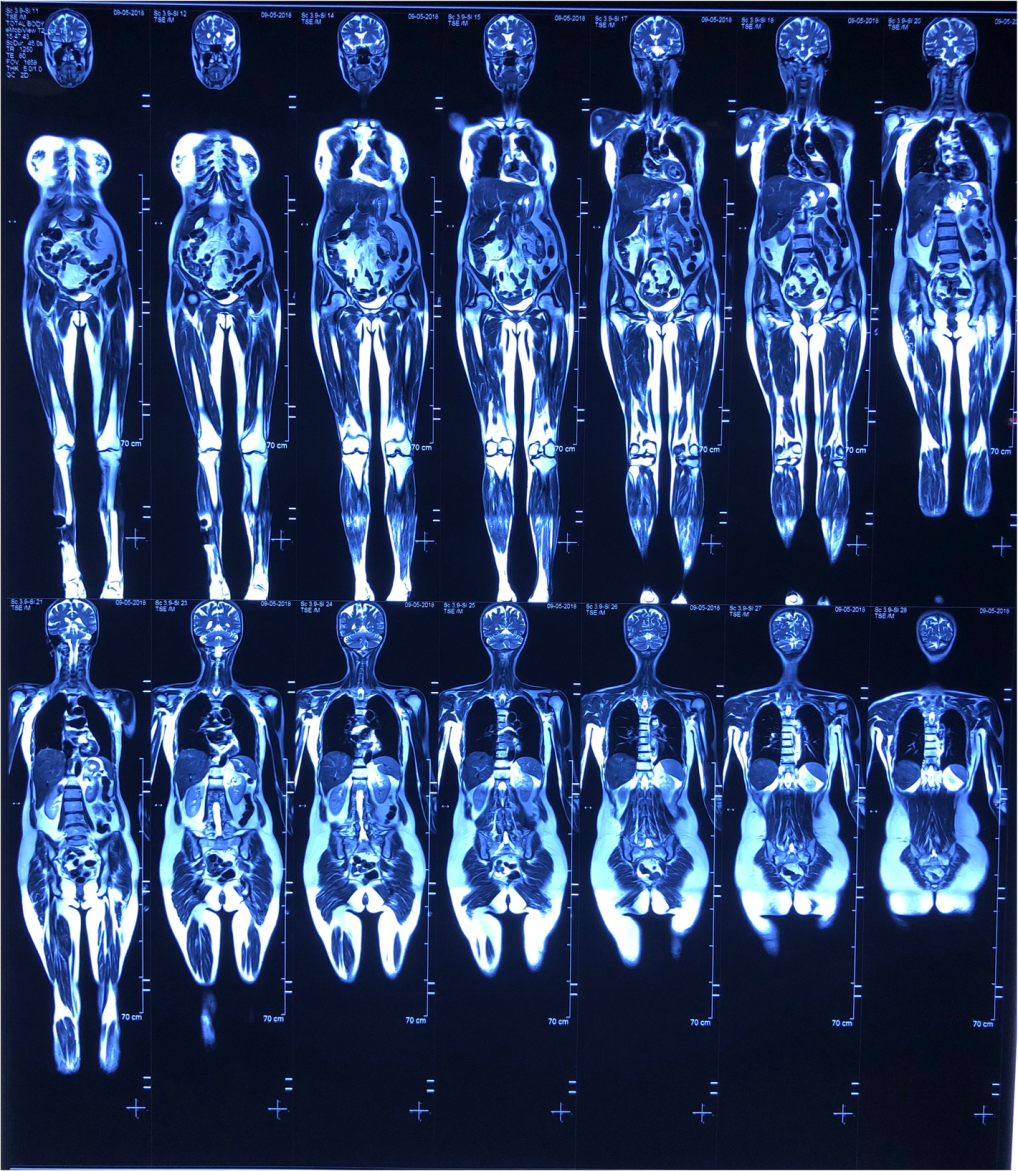
Normal whole-body magnetic resonance imaging

Skin biopsies from four body sites showed normal epidermis and thickened reticular dermis with swollen collagen bundles separated from one another by clear spaces, resulting in fenestration. The skin appendages were not atrophied or bound down. Alcian blue staining showed interstitial mucin deposition suggestive of scleredema. Serum protein electrophoresis demonstrated an abnormal monoclonal band in the gamma region with a paraprotein level of 8.9 g/dl. Immunofixation showed an abnormal band in the gamma region consisting of IgA and κ. Bone marrow biopsy revealed abnormal monoclonal plasma cells (15%) with multinuclearity. There was no evidence of end organ damage with normal calcium, renal function, and full blood count, and whole-body magnetic resonance imaging did not reveal any evidence of bone involvement (Fig. 3). The patient’s diagnosis was revised as scleredema type 2 associated with IgA-κ smoldering myeloma. She was commenced on intravenous immunoglobulin (IVIG) monthly (1 g/kg for 2 days per month), and a hemato-oncologist started intravenous bortezomib cycles (1.7 g on day 1, day 8, day 22, and day 29). Currently, she was receiving 6 months of IVIG and four cycles of intravenous bortezomib, and significant improvement of the skin was observed.

## Discussion

In this report, we present a case of a patient with widespread thickening of the skin predominantly involving the trunk and proximal extremities for more than 30 years who was misdiagnosed with systemic sclerosis. Absence of Raynaud’s phenomenon; sclerodactyly; characteristic lung, gastrointestinal, and cardiac involvement of systemic sclerosis; and repeatedly negative antinuclear antibody test results led to reevaluation of the diagnosis. Later she was diagnosed with scleredema with smoldering myeloma, and she responded well to treatment.

In keeping with the literature, even though scleredema with monoclonal gammopathy is reported, multiple myeloma (MM)-associated scleredema is rare. In 1974, Korting *et al.* reported one patient with MM; in 1984, Venencie *et al*. reported one patient with smoldering myeloma; and in 1987, Ohta *et al*. reported one patient with MM [2–4]. After that, several case reports were reported (Table 2). Seven were male patients, six were female, and the majority were above the age of 50. Interestingly, one patient was in his 20s [5]. Only two patients were reported with smoldering myeloma (one with IgG-κ and the other with IgG-λ) and scleredema [2, 6] in the literature, and all the others had MM (five with IgG-κ, four with IgA-κ, one with IgG-λ, and one with IgA-λ; two had IgG and the light chain was not mentioned). This shows that the number of patients with IgG and IgA were observed in almost equal numbers and that the majority of patients had κ-light chains compared with λ-light chains (10 patients with κ, 3 patients with lambda). Even in scleredema associated with monoclonal gammopathy without MM, IgG-κ predominates (10 of 23 cases in one review) [7]. Our patient is the first to be reported with scleredema who was diagnosed with smoldering myeloma of IgA-κ.

**Table 2:** Literature review of patients with myeloma and scleredema

Year	Age (years)	Sex	Clinical features	Myeloma	Bone marrow plasma cells	Myeloma investigations	Treatment	Prognosis	Reference
1974	37	M	2 years of disease	MM (IgG-κ)		Urine Bence-Jones-positive	Chemotherapy	Good response	[3]
1984	69	M		Smoldering myeloma (IgG-κ)					[2]
1987	64	M	Rapid onset of skin involvement of face and chest; 7 years of little change; rapid progression to involvement of the shoulders, arms, back, abdomen, and thighs over 2 years	MM (IgG-κ); IgG concentration of 4500 mg/dl		Urine immunoelectrophoresis IgG-κ monoclonal proteinuria, anemia; skeletal survey normal	Prednisolone 80 mg/day and melphalan 14 mg/day, given for 4 days and repeated monthly	From the sixth cycle, clear clinical evidence of softening of the skin and improved joint mobility	[4]
1988	72	F	Symmetrical woody induration of face, neck, chest, shoulders, upper back, chest, abdomen, thigh over 2 years	MM (IgG-κ); IgG concentration of 2900 mg/dl		Osteolytic lesions, Bence-Jones-negative	Melphalan	Recovery in 1.5 years	[14]
1988	76	F	Stiffening of the skin of upper trunk, arms, neck, face over 24 h with woody induration	MM (IgA-κ); IgA 814 mg/dl	20%	Urine κ-light chains, multiple lytic lesions on skull x-ray	IV cyclophosphamide 750 mg over 3 days, prednisolone 30 mg daily for 3 days; six pulses; each pulse over interval of 3 weeks	Complete recovery of scleredema and remission of MM	[15]
1988	62	F	Thickening of skin over 23 years with induration over face, neck, thorax, arms with loss of movements of the underlying joints	MM (IgG-λ); IgG 2500 mg/dl	40%	anemia, urine Bence-Jones λ-light chains, cardiomyopathy	Pulse chemotherapy	Death with sepsis	[9]
1992	46	M	Stiffening of face, neck, back, shoulders, chest, arms, hands, fingers with woody induration	Smoldering myeloma (IgG-λ)	28%				[6]
1995	74	M	Marked induration of the skin of neck, shoulders, upper chest, back, and upper arms over 15 years	MM; IgA-κ; IgA, 2530 mg/dl	67%	Anemia, Bence-Jones protein *(*κ-light chain), osteolytic lesions	Chemotherapy with intravenous vincristine, cyclophosphamide, oral melphalan, prednisone 90 mg; six cycles at 3-weekly intervals	After the sixth chemotherapy cycle, myeloma was in remission; marked improvement of the skin	[7]
1997	56	F	Scleredema over the last 6 years with acanthosis nigricans	MM; IgA-κ			Melphalan and prednisolone	Recovery	[10]
2000	63	F	thickening of the skin on the face, neck, shoulders, arms, and upper torso while receiving treatment for MM	MM; IgG-κ; IgG 9600 mg/dl	80%	Urine IgG-κ light chain protein, anemia, lytic lesions in the skull	Melphalan 10 mg/day, cyclophosphamide 200 mg/day, prednisolone 60 mg/day given for 5 days, vincristine 2 mg given on the first day; six courses with a 3-week gap	Softening of the skin observed from sixth treatment cycle	[8]
2001	70	F	over 12 months of period, she had developed a progressive induration and stiffness of the skin of her face, neck, shoulders, and upper aspect of her arms	MM; IgA-λ; IgA, 1830 mg/dl	38%	Urine Bence-Jones protein	Oral melphalan, oral prednisone; six pulses, each pulse at an interval of 1 month	Clinical evidence of softening of the involved skin observed	[16]
2008	28	M	Eight years of progressive diffuse cutaneous thickening of face, trunk, arms, and thighs	MM; IgA-κ	55%	Anemia, myelofibrosis	Thalidomide, dexamethasone	Improvement in the texture of the skin	[5]
2013	60	M	Symmetric, nonpitting swelling of the face, neck, trunk, and upper extremities	MM; IgG-κ	10%	Multiple osteolytic lesions	IV cyclophosphamide, bortezomib, dexamethasone; six cycles and autologous stem cell transplant	Skin induration gradually decreased during treatment with complete recovery	[12]
2015	62 (two patients)		Generalized symptomatic scleredema	IgG MM			IV cyclophosphamide, bortezomib, dexamethasone, and IVIG	Significant improvement	[13]

*Abbreviations: IgG* Immunoglobulin G, *IV* Intravenous, *IVIG* Intravenous immunoglobulin, *MM* Multiple myeloma, *M* male, *F* female

The diffuse woody induration described in almost all the cases in the literature involved the face, neck, back, shoulders, chest, and upper arm. Similar to our patient, all the patients had skin manifestations for a long time before the diagnosis of myeloma, except in one case where the skin changes appeared while the patient was receiving treatment for myeloma [8]. As in our patient, none of the patients in the literature had Raynaud’s phenomenon; sclerodactyly; or characteristic lung, gastrointestinal, or cardiac involvement of systemic sclerosis, which is important to differentiate from systemic sclerosis. One case report described a patient with MM and scleredema who developed cardiomyopathy. Deposition of acid mucopolysaccharide in the heart is proposed as the mechanism for this scleredema cardiomyopathy [9]. Acanthosis nigricans [10] and myelofibrosis [5] are also described in patients with scleredema and MM.

The possible pathology of monoclonal gammopathy and scleredema is still not clear. Kovary *et al*. suggested that paraproteins may function as antibodies directed against connective tissues, but monoclonal immunoglobulins were not detected in the skin by direct immunofluorescence microscopy [11]. This is in contrast to scleromyxedema (lichen myxedematosus), from which scleredema can be distinguished clinically and histologically [11]. Ohta *et al*. showed that serum from patients with scleredema stimulates collagen production in normal skin fibroblast cultures, collagen production in autologous cell cultures, and sulfate incorporation into fibroblasts [4]. They suggested that circulating serum factors in these patients, possibly related to the paraproteins, may stimulate the synthesis of extracellular macromolecules by dermal fibroblasts, leading to dermal fibrosis. On the basis of these studies, we can postulate that immunological factors may play a role in the pathogenesis of scleredema.

Interestingly, all the patients in the literature showed improvement of the skin condition with therapy. Different chemotherapy regimens, including melphalan, cyclophosphamide, vincristine, and thalidomide combined with steroids, were used in these cases (Table 1). A bortezomib-based regimen has also shown a convincing response [12, 13]. In the two case reports with scleredema associated with smoldering myeloma, we were unable to find any specific therapy given for the skin condition. However, IVIG has shown significant skin condition improvement in two patients with scleredema [13]. Grudeva-Popova and Dobrev suggested that noninvasive skin elasticity measurements can be used to assess improvement after treatment [8].

## Conclusions

We highlight that scleredema should be considered in the differential diagnosis of patients with diffuse skin thickening without characteristic features of systemic sclerosis. In these patients, it is also important to investigate for monoclonal gammopathy and myeloma. Even if the initial screening result is negative, serum protein electrophoresis should be performed at regular intervals because paraproteinemia may appear later and, when present, may progress to myeloma. This case report and others in the literature show that this condition is treatable with significant improvement of the skin condition.

## Abbreviations

ALT: Alanine aminotransferase
AST: Aspartate aminotransferase
IgG: Immunoglobulin G
INR: International normalized ratio
IV: Intravenous
IVIG: Intravenous immunoglobulin
MM: Multiple myeloma
WBC: White blood cells
